# Diagnosis of Acute Isolated Sphenoid Sinusitis Based on a Positive Kernig Sign in a Pediatric Patient

**DOI:** 10.7759/cureus.93291

**Published:** 2025-09-26

**Authors:** Hinako Yamamoto, Takateru Ihara

**Affiliations:** 1 Department of Pediatrics, Hyogo Prefectural Amagasaki General Medical Center, Amagasaki, JPN

**Keywords:** endoscopic sinus surgery (ess), isolated sphenoid sinusitis, kernig sign, meningeal irritation, pediatric headache

## Abstract

Acute isolated sphenoid sinusitis in children is relatively rare, often presents with atypical symptoms, and may result in intracranial complications; therefore, early diagnosis is essential. We report the case of a 14-year-old girl who presented with a headache and a positive Kernig sign. Computed tomography (CT) demonstrated fluid retention in the sphenoid sinus, and lumbar puncture showed no abnormalities in the cerebrospinal fluid (CSF). Based on these findings, she was diagnosed with acute isolated sphenoid sinusitis without intracranial complications. However, the patient showed a poor response to antibiotic therapy, and magnetic resonance imaging (MRI) revealed dural thickening, necessitating surgical intervention. Intraoperatively, bony destruction and CSF leakage were identified, suggesting extension of inflammation into intracranial structures. Following treatment, the patient’s Kernig sign resolved. To our knowledge, there have been no previous reports explicitly describing the appearance of the Kernig sign in sphenoid sinusitis. This case provides important insights into the clinical significance of this finding.

## Introduction

Acute isolated sphenoid sinusitis in children is an uncommon entity [1]. The most common symptom is headache, but its location varies [2]. Fever occurs in approximately 50% of cases and rhinorrhea in only 20%, often resulting in atypical clinical presentations [3]. Ocular manifestations are considered specific findings due to the anatomical proximity of the orbit to the sphenoid sinus [2].

Because acute isolated sphenoid sinusitis can cause serious intracranial or orbital complications such as orbital cellulitis, sepsis, meningitis, epidural abscess, and subdural abscess, early diagnosis and appropriate treatment are essential [4]. Computed tomography (CT) and magnetic resonance imaging (MRI) are useful diagnostic tools, and surgical drainage is indicated when antibiotic therapy is ineffective or neurological abnormalities are present [3]. The appearance of meningeal signs is an important indicator of intracranial complications, and past reports have noted neck stiffness [3-5]. However, the clinical significance of the Kernig sign in sphenoid sinusitis has not been addressed.

We present a pediatric case of acute isolated sphenoid sinusitis requiring surgical intervention in which the Kernig sign was observed despite normal cerebrospinal fluid (CSF) findings. This case offers an opportunity to reconsider the significance of the Kernig sign.

## Case presentation

A previously healthy 14-year-old girl presented to the emergency department with headache, fever, and vomiting. She had developed a left temporal headache five days earlier, which became bilateral and persistent two days before presentation. The headache was severe enough to interfere with daily life and was accompanied by non-bloody, non-bilious vomiting, photophobia, and a fever of 38.1°C. There were no upper respiratory symptoms such as cough, nasal obstruction, or rhinorrhea. She had not received any antibiotics prior to presentation.

On initial examination, vital signs showed a temperature of 38.1°C, a heart rate of 100 beats per minute, blood pressure of 112/64 mmHg, and SpO₂ of 95% on room air. She was alert with a Glasgow Coma Scale (GCS) score of E4V5M6. Neurological examination revealed a positive Kernig sign when lying supine with the hip and knee flexed; passive knee extension was limited beyond 135° with resistance (Video 1). There was no history of musculoskeletal disorders that could cause limitation of knee extension. Tenderness was noted over the frontal and maxillary sinuses, but there was no nuchal rigidity, diplopia, ocular motility disturbance, or visual impairment. No other neurological abnormalities were observed.

**Video 1:** Positive Kernig sign on the day of presentation Neurological examination revealed a positive Kernig sign. When lying supine with the hip and knee flexed, passive knee extension was limited beyond 135° with resistance.

**Figure 1:**
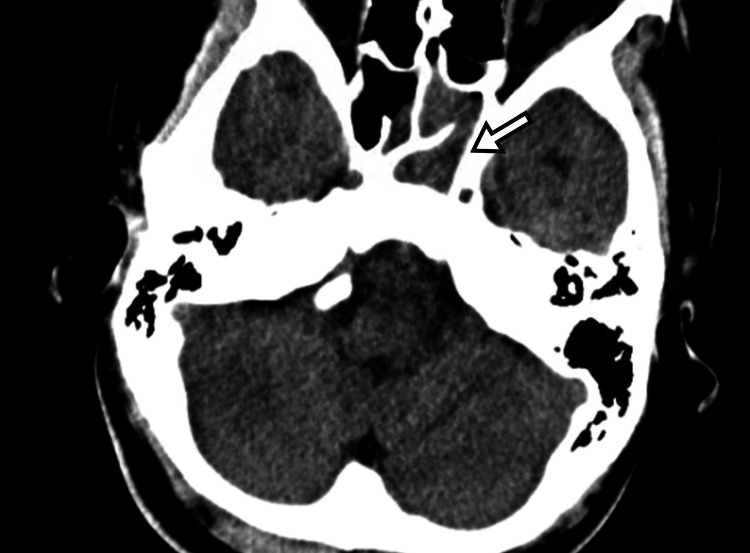
Head computed tomography (CT) on the day of presentation demonstrated fluid retention in the left sphenoid sinus (arrow).

Laboratory studies showed leukocytosis with a white blood cell (WBC) count of 14,100/μL (neutrophils 89.6%) and an elevated C-reactive protein (CRP) level of 11.04 mg/dL (Table 1). Head CT demonstrated fluid retention in the left sphenoid sinus (Figure 1). Given the positive Kernig sign but lack of other evidence for bacterial meningitis, she was diagnosed with sphenoid sinusitis based on history and imaging, started on oral antibiotics, and discharged for outpatient follow-up.

**Table 1:** Laboratory findings on the day of presentation

Laboratory Test	Result	Reference Value	Unit
White blood cell (WBC) count	14,100	4,000–10,000	/µL
Neutrophils	89.6	40–70	%
C-reactive protein (CRP)	11.04	<0.3	mg/dL
Cerebrospinal fluid (CSF) cell count	1	0–5	/µL
CSF glucose	74	50–80	mg/dL
CSF protein	19.8	15–45	mg/dL

Six hours later, she returned to the emergency department with a worsening headache. Her temperature was 38.7 °C, heart rate 124 beats/min, and her GCS score remained E4V5M6. The Kernig sign persisted. Because of the persistent Kernig sign and severe headache, a lumbar puncture was performed. CSF analysis was normal, with 1 cell/µL, a CSF glucose level of 74 mg/dL, and a total CSF protein level of 19.8 mg/dL (Table 1). Considering the possibility of early meningitis or meningeal irritation without meningitis, intravenous antibiotics at meningitis doses were initiated.

**Figure 2:**
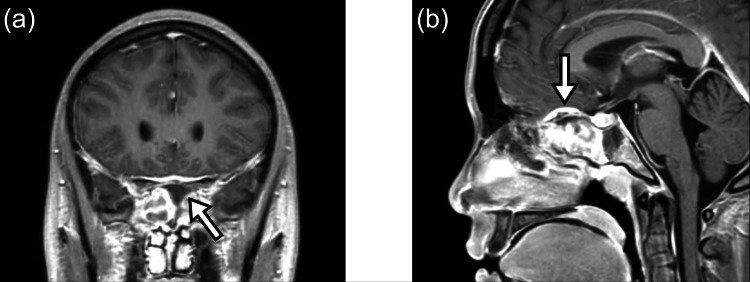
Contrast-enhanced magnetic resonance imaging (MRI) on day 5 (a) Marked mucosal thickening, enhancement, and fluid retention in the left sphenoid sinus (arrow); (b) Localized dural thickening near the anterior cranial base (arrow), suggesting inflammatory extension.

On the following day, blood cultures grew *Haemophilus influenzae*. CSF culture was negative. Fever persisted until hospital day 4. Contrast-enhanced MRI on day 5 revealed marked mucosal thickening, enhancement, and fluid retention in the left sphenoid sinus (Figure 2a). Localized dural thickening was observed near the anterior cranial base, suggesting the possibility of inflammatory extension (Figure 2b). Bony destruction of the sphenoid sinus was also identified; however, no cavernous sinus thrombosis or brain abscess was detected.

Emergency endoscopic sinus surgery was performed the next day, as the patient developed a recurrent fever on day 6 along with a persistent headache. Intraoperatively, copious foul-smelling yellowish-white pus was drained, which was sent for culture and yielded α-hemolytic and coagulase-negative staphylococci (CNS). The sphenoid sinus mucosa was markedly swollen, erythematous, and hemorrhagic. Pulsatile clear fluid leakage was observed from the posterior-superior wall of the left sphenoid sinus, raising suspicion of CSF leakage.

Postoperatively, intravenous cefotaxime was administered at a dosage of 3 g every eight hours for three weeks, consistent with the management of epidural abscess. Her fever and headache gradually improved without recurrence. By day 13 of treatment, the Kernig sign had disappeared, suggesting that the preoperative finding was a pathological response secondary to sphenoid sinusitis (Video 2).

**Video 2:** Resolution of the Kernig sign after treatment By day 13 after the initiation of treatment, the Kernig sign had become negative.

On day 19, a follow-up contrast MRI showed improvement of dural thickening but new findings suggestive of sphenoid sinus osteomyelitis. Oral amoxicillin 1500 mg/day in three divided doses was therefore continued for an additional five weeks, making the total treatment duration eight weeks. At the end of therapy, an MRI showed no abscess formation, and the subsequent course was favorable.

## Discussion

Sphenoid sinusitis is rare among paranasal sinus infections [6]; however, its anatomical proximity to critical structures such as the skull base, dura mater, cavernous sinus, and cranial nerves predisposes patients to intracranial complications, including meningitis, subdural abscess, brain abscess, and optic neuropathy [4]. Thus, early risk recognition and careful monitoring are required.

When meningitis is suspected, lumbar puncture is indicated; however, meningeal signs are not always accompanied by abnormal CSF findings [7]. The Kernig sign is one of the meningeal signs [8]. In this case, nuchal rigidity was absent, whereas the Kernig sign was positive. While the Kernig sign has high specificity but low sensitivity and is considered an adjunctive diagnostic finding in meningitis, it is a standardized, reproducible physical examination maneuver [9].

Kernig sign is considered positive when, in the supine position with the hip and knee flexed at 90°, the knee cannot be extended beyond 135° due to resistance [8, 10]. In children, the coexistence of musculoskeletal disorders that limit knee extension is rare, making the assessment more objective and reliable [8]. In this case, although CSF findings were normal and no other neurological abnormalities were present, the persistence of the Kernig sign prompted reevaluation for intracranial disease and led to a change in treatment strategy.

The Kernig sign is not specific to meningitis and is best interpreted as a pain-avoidant response to neural tissue stretch. It has been reported in various conditions, including subarachnoid hemorrhage, epidural hemorrhage, and epidural abscess [11,12]. During knee extension in a flexed-hip position, mechanical tension is first applied to the sciatic nerve and can be transmitted proximally along the neuroaxis to the lumbosacral plexus and the nerve roots and, as a result, along the entire length of the spinal cord. Therefore, in this patient, focal meningeal inflammation related to sphenoid sinusitis may have interacted with the stretch applied during testing so that pain was provoked and a protective flexion response was elicited, yielding a positive Kernig sign.

An early phase of meningitis is also possible. Although no overt intracranial complications such as brain abscess or subdural abscess were identified, MRI showed focal dural thickening at the anterior cranial base, and intraoperative findings revealed sphenoid bone destruction with pulsatile CSF leakage, findings that indicate spread of inflammation to intracranial structures with disruption of anatomical barriers. In this context, the positive Kernig sign may have represented an early manifestation of meningitis, that is, early intracranial extension, at a time when CSF studies were still normal [2].

Previous reports have described cases of sphenoid sinusitis presenting with meningeal signs in the absence of intracranial complications, but these have referred only to neck stiffness [3-5]. To our knowledge, no prior case has explicitly documented the presence of the Kernig sign. Therefore, this case may represent the first report to demonstrate the clinical significance of the Kernig sign in sphenoid sinusitis. The presence of the Kernig sign may indicate not only meningitis but also deep-seated infection with extension to the bone and dura.

## Conclusions

Due to its anatomical characteristics, sphenoid sinusitis requires early imaging and therapeutic intervention even in the absence of overt intracranial disease. Particularly, when meningeal signs or intractable headache persist despite normal CSF findings, evaluation with CT or MRI is essential. In this case, imaging and intraoperative findings were decisive for management, and surgical intervention led to a favorable outcome.
